# Outcomes of double level osteotomy for osteoarthritic knees with severe varus deformity. A systematic review

**DOI:** 10.1051/sicotj/2022009

**Published:** 2022-04-01

**Authors:** Hany Elbardesy, André McLeod, Hazem S. Ghaith, Samir Hakeem, Philip Housden

**Affiliations:** 1 Department of Trauma and Orthopaedics, East Kent University Hospital Kent TN240LY UK; 2 Orthopaedic Senior House Officer, Department of Trauma and Orthopaedic Surgery, Cork University Hospital Wilton, Cork T12DC4A Ireland; 3 Faculty of Medicine, Al-Azhar University Cairo 11651 Egypt; 4 Orthopaedic Registrar, Department of Trauma and Orthopaedic, Galway University Hospital Galway H91 YR71 Ireland

**Keywords:** Knee, Osteoarthritis, Double level osteotomy, Osteotomy, Varus knee

## Abstract

*Background*: When correcting severe genu varus deformity, knee surgeons must choose between performing a single or double-level osteotomy. This systematic review aims to provide this equipoise with some clarity. *Patients and methods*: We conducted this study following the Preferred Reporting Items for Systematic Reviews and Meta-analyses Statement (PRISMA) and the Cochrane Handbook for systematic reviews and meta-analysis. Studies evaluating the effect of the double level osteotomy (DLO) or those comparing it to high tibial osteotomy (HTO) from all regions and written in any language were included. *Results*: Six studies were included in this systematic review. They were prepared and analysed using Review Manager V5.0 [Computer Program] (RevMan5). Performing DLO resulted in restoring patellar height, joint-line convergence angle (JLCA), and mMPTA to normal values. DLO was also more successful at avoiding joint line obliquity (JLO) in severe varus deformity when compared to HTO (*P* < 0.001). No significant difference was reported between the two cohorts regarding the mLPTA. DLO resulted in satisfactory short term KOOS and IKDC scores. The complication rate after DLO was 2.28%. *Conclusions*: DLO showed a low complication rate and satisfactory short term KOOS and IKDAC scores. Randomised control trials with long term follow-up comparing the DLO and HTO are recommended.

## Introduction

Corrective osteotomy of genu varus is a valuable treatment option for active young patients with Unicompartmental Osteoarthritis (UCOA) of the knee [1–4]. The High Tibial Osteotomy (HTO) has shown to be effective in treating mild varus deformity with satisfactory midterm outcomes [5, 6]. Despite its efficacy in mild varus deformity, some authors have reported negative outcomes for HTO in severe varus deformity [7–10]. The HTO correction of severe varus deformity involves cutting a large opening wedge. Removal of this large wedge leads to the abnormal inclination of the lateral tibial plateau and subsequently results in the cartilage of the knee joint experiencing a high sheer force [6, 8]. Moreover, HTO can result in abnormal femoral subluxation and straining of the joint capsule, making future Total Knee Arthroplasty (TKA) a challenging procedure [11–16]. As a result of these complications, double level osteotomy (DLO) has been advocated for severe genu varus deformity to restore normal joint alignment [17]. Multiple studies published more than 30 years ago advocated against the DLO, however, these studies often included patients with rheumatoid knees [18–23]. The surgeons of the time also failed to consider restoration of the mechanical axis of the knee and lacked the detailed preoperative planning associated with DLO today. As such, the advantage of the DLO over the HTO was not widely accepted by the orthopaedic surgeons of that era. The DLO technique has evolved over the last twenty years, and its popularity is steadily growing, particularly in countries like Germany and Japan [24, 25]. Severe genu varus can be defined as having a mechanical Medial Proximal Tibial Angle (mMPTA) of more than 95° [26, 27], or in some studies, of more than 93° [24]. Other authors defined it as a mechanical tibiofemoral varus angle (mFTA) of ≥ 3° [28, 29]. Some authors have also measured the mechanical Distal Femorotibial Angle (mDFTA) and considered an angle of more than 90° as another indication for DLO [7, 30–32]. The degree of varus deformity can also be broadly categorised into “mild” (3°–5°), “moderate” (6°–8° varus), or “severe” (≥ 9°) [33].

Despite the abundance of literature examining genu varus, there is still a lack of data with regard to the outcomes and efficacy of the DLO as a treatment for genu varus. This study aims to systematically review the outcomes and efficacy of DLO compared to HTO when used in treating knee osteoarthritis with genu varus deformity. The radiological outcomes of patellar height, mechanical medial proximal tibial angle (mMPTA), mechanical lateral proximal tibial angle (mLPTA), and joint-line convergence angle (JLCA) will be evaluated side by side with patient-reported outcomes measures (PROM) such as Knee Injury and Osteoarthritis Outcome Score (KOOS), International Knee Documentation Committee (IKDC). Reported complications of the DLO will also be evaluated.

## Materials and methods

We performed this study following both the Preferred Reporting Items for Systematic Reviews and Meta-analyses Statement (PRISMA) and the Cochrane Handbook for systematic reviews and meta-analysis [34]. We conducted an initial search using MEDLINE-OVID, Web of Science, PubMed, EMBASE-OVID, Google Scholar, and Cochrane Library. We used the following keywords and their combinations: Double osteotomy, High tibial osteotomy, and Genu varus. Articles published from 2000 to July 2021 were included in our literature search and were limited to studies in human subjects published in any language. We also cross-referenced the citations of recovered articles to ensure that all relevant studies were captured.

### Study selection criteria

All forms of comparative studies which involved patients undergoing DLO or HTN for severe genu varus deformity were included (retrospective/prospective cohorts, RCTs). We excluded all studies published before 2000 as all these studies did not exclude the rheumatoid knees. Moreover, the detailed preoperative planning and restoration of the mechanical axis of the knee were not accurately considered in that days [18–23]. We excluded cadaveric studies, conference abstracts, letters to the editor, and reviews.

### Data extraction and analysis

Eligible titles and abstracts identified by the initial search were screened independently by four authors to assess their eligibility for inclusion in the systematic review. Each manuscript was then fully screened by the authors, and a final eligibility assessment for all included studies was performed. Data extraction was completed, and any discrepancies found were resolved by discussion between all reviewers. Collected information included the lead author, year published, publishing journal, country of study, level of evidence, study design, number of centres, study length, number of participants, age, gender, and Body Mass Index (BMI).

### Outcome measures

The radiological outcomes of interest were patellar height, mMPTA, mLPTA, and JLCA. The clinical PROMs were KOOS, IKDC, and complications of the DLO.

## Results

### Study characteristics

**Figure 1:**
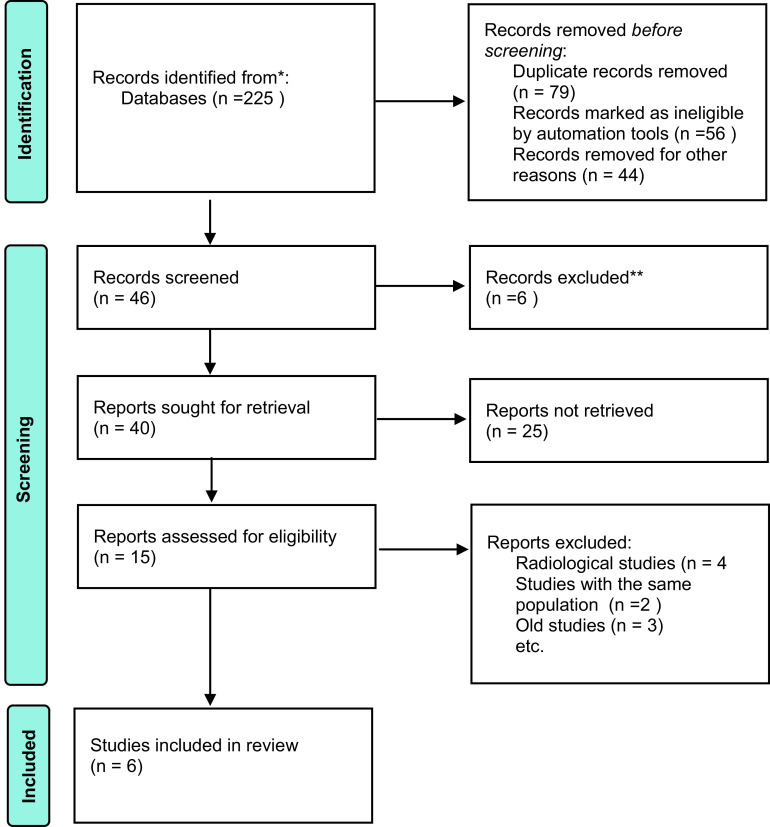
Preferred reporting items for systematic reviews and meta-analyses (PRISMA) flow chart.

Our literature review returned 125 articles when excluding duplicates. Screening of the titles and abstracts revealed 46 articles that were eligible for full-text screening. Thirty-seven articles were then removed for not meeting selection criteria, subsequently leaving six articles that were eligible for quantitative review. A flow chart demonstrating the study selection process is provided (Figure 1). A summary of the characteristics of included papers is provided (Table 1).

**Table 1:** Study’s characteristics.

Study	Journal	Type of study	Level of evidence	Numbers of DLO	Numbers of MHTO
Akaoka et al. [35]	ASMART	RCS (double arm)	II	26	26
Nakayama et al. [16]	Knee Surgery, Sports Traumatology, Arthroscopy	Retrospective case series	IV	20	NA
Babis et al. 2002	JBJS	Prospective case series	NA	24	NA
Hai et al. [37]	Progress in Rehabilitation Medicine journal	Retrospective case series	NA	26	NA
Saragaglia et al. [40]	International Orthopaedics (SICOT)	Retrospective case series	NA	38	NA
Schröter et al. [24]	AOTS	Retrospective case series	IV	33	NA

ASMART: Asia-Pacific Journal of Sports Medicine, Arthroscopy, Rehabilitation and Technology, AOTS: archives of orthopaedic and trauma surgery, JBJS: The Journal of Bone and Joint Surgery.

### Patient baseline characteristics

Our review included a total of 175 knees. The average age of the cohort was 55.9 years (± 5.85 years). Sixty-eight patients (38.85%) were female, and 107 were male (61.15%), and the cohort had an average BMI of 28.12 kg/m^2^ (± 2.94). A table summarising the patient demographics is included (Table 2).

**Table 2:** Patient demographics.

Study	Gender	Age DLO	Age MHTO	PROM	Height	Follow-up months	BMI
Akaoka et al. [35]	3 M, 23 F, (3 M/23 F)	62.3 (6.8)	62.3 (5.6)	HKA angle, mLDFA, mMPTA, Modified Insall-Salvati Index, Modified Caton-Deschamps Index, Modified Blackburne-Peel Index, Lateral patellar tilt, Lateral patellar shift	1.57 (7.6), 1.57 (7.1)	35.5 (8.0), 39.0 (4.9)	24.6 (2.5), 24.5 (2.7)
Nakayama et al. [16]	5m, 15 F, (5 M/15 F)	62.5 (6.8)	NA	mTFA, mLDFA, mMPTA, and JLCA,KOOS and IKDC	NA	20.7 (6.8)	NA
Babis et al. [7]	22 M, 2 F, (22 M/2 F)	50	NA	Mechanical tibiofemoral angle (mTFA), Mechanical deformity, Medial plateau force distribution, Range of joint-line obliquity, Collateral ligament tension	1.74	82.7	30.1
Hai et al. 2020	15 M, 11 F, (15 M/11 F)	60.2 (4.7)	NA	JKOM, JOA, Range of knee extension, Range of knee flexion, Total arc of range of motion of knee, Hip–knee angle, and α angle	159.7 (7.0)	6	26.7 (2.9)
Saragaglia et al. [40]	29 M, 9 F, (29 M/9 F)	50.9 (7.1)	NA	Lyshölm-Tegner score, KOOS score, HKA angle, MPTMA, MDFMA.	1.71	46 (27)	29.3 (4.3)
Schröter et al. [24]	23 M, 5 F (23 M/5 F)	50 (9.7)	NA	mTFA, mLDFA, MPTA, and Lequesne-, Lysholm-, Oxford-, and IKDC-score	NA	18	29.9 (5.0)

SD: standard deviation, NA: Not applicable, BMI: Body Mass Index.

## Systematic review

### Radiological results

#### Patellar height

Two studies reported on the patellar height. Akaoka et al. [35] did not report any significant difference between the two cohorts with regard to patellofemoral biomechanics. However, Fürmetz et al. [36] reported a significant reduction in the patellar height for the HTO group compared with the DLO cohort.

#### Mechanical medial proximal tibial angle (mMPTA)

Four studies reported on mMPTA. Feucht et al. [33] analysed radiographs of 303 knees in order to determine the ideal osteotomy level to avoid JLO. About 63% of knees required a DLO to correct mMPTA, while in 12% HTO was appropriate for correction. However, when allowing for 2 degrees of overcorrection, the percentage of HTOs significantly increased to 57% of knees while 33% required DLO. Other studies reported DLO showed improved restoration of mMPTA to normal values while avoiding JLO when compared with HTO in severe varus deformity (*P* < 0.001) [7, 24, 36].

#### Mechanical lateral proximal tibial angle (mLPTA)

Three studies compared postoperative mLPTA between the DLO and HTO cohorts. No significant difference was reported between the two cohorts [33, 35, 36]. Other non-comparative studies reported satisfactory postoperative mLPTA alignment after DLO [25, 37–39].

#### Joint-line convergence angle (JLCA)

Four studies reported on the JLCA. All four found that DLO corrected JLCA more successfully than HTO in severe varus deformity (*P* < 0.05) [16, 33, 37, 39].

### Clinical outcome

#### Knee Injury and Osteoarthritis Outcome Score (KOOS)

Two studies reported on KOOS. They both reported a significant improvement in KOOS at six, twelve, and 46 months after DLO (*P* < 0.05) [16, 40].

#### International Knee Documentation Committee (IKDC) score

Three studies reported on the IKDC score. DLO resulted in a significant improvement in IKDC at six and twelve months compared to mean preoperative IKDC score (*P* < 0.05) [7, 16, 24].

### Complications

Four out of 175 patients (2.28%) suffered complications after DLO. One patient sustained an injury to the popliteal artery during the distal femoral osteotomy [16]. Another patient developed a wound infection three months postoperatively and was successfully treated with oral antibiotics [37]. One patient suffered a fracture of the medial hinge after distal femur osteotomy and subsequently required revision surgery [24]. The fourth patient developed postoperative valgus deformity due to collapse of the supracondylar part of the DLO. This was due to inadequate fixation [7] Table 3.

**Table 3:** Complications.

Study	Complications	Numbers of DLO	Percentage
Akaoka et al. [35]	None	26	0%
Nakayama et al. [16]	A major intraoperative arterial injury was encountered in a 44-year-old female patient. In this case, the popliteal artery was injured during the distal femoral osteotomy.	20	5%
Babis et al. [7]	In one knee, the supracondylar part of the double osteotomy collapsed into excessive valgus one week postoperatively because of insufficient fixation/ The two undercorrected knees had a 74% and 87% residual medial overload	24	4.17%
Hai et al. [37]	One case of wound infection at the tibial site was diagnosed 3 months postoperatively and was successfully treated with oral antibiotics.	26	3.85%
Saragaglia et al. [40]	No	42	0%
Schröter et al. [24]	One patient had a complication with a fracture of the medial hinge after distal femur osteotomy; this resulted in an overcorrection, and the problem was solved with revision surgery.	37	2.7%
Total	4	175	2.28%

## Discussion

The most important findings in this study are the relatively low complication rate of the DLO and the satisfactory postoperative KOOS and IKAC scores. A 3D finite element analysis study reported high shear stress at the medial tibial plateau following correction of more than 5° using HTO [16]. Thus, some authors advocated using a DLO to correct any varus deformity with mMPTA more than 95° [16, 41, 42]. Furthermore, Schuster et al. [29] reported unfavourable outcomes at ten years follow-up in 73 patients with mMPTA > 95° who underwent HTO. Overcorrection of varus deformity with HTO failed to change the outcome at two years follow-ups [43]. Of note, an mMPTA of 95° with a JLO of 5° is not an indication for correction as it is compensated by changes in both the ankle and hip joints [44–46]. Some authors advocate against the DLO as it requires delayed weight-bearing after the surgery, however, a recent study proved that the rehabilitation after DLO can start on day 3 post-surgery with the achievement of full weight-bearing after 4 weeks [37]. The favourable outcomes of DLO, when compared to HTO, stem from multiple factors. In advanced genu varus, the lateral compartment of the knee is usually pristine due to the preoperative unloading. Additionally, DLO results in less tension on the knee’s ligaments due to equal distribution of loads over the whole knee after deformity correction. Furthermore, the DLO corrects the varus deformity of the knee without residual JLO, subsequently decreasing the shear stress at the lateral femoral condyle and changing the load over the medial compartment by 5% [16, 47]. Of note, the recent less invasive techniques in DLO have reduced morbidities and the theoretical disadvantage of postoperative decrease in the arc of movement [19]. Feucht et al. [33] reported that with overcorrection of the varus deformity, 65% of patients could be corrected with a single osteotomy, either HTO (57%) or DFO (8%). Only 33% of patients with severe varus deformity require DLO.

Babis et al. [7] reported the indication and outcomes of the DLO for advanced varus deformity. They also performed a postoperative computerised assessment using an Osteotomy Analysis and Simulation Software (OASIS). They reported satisfactory survival at 8.33 years follow-up with a failure rate of 5%. A similar technique used preoperative computerised templating and intraoperative navigation system to determine the desired amount of correction and was reported by other surgeons as having an excellent outcome [7, 38]. Thus, the surgical technique is a keystone for satisfactory outcomes. The use of a rigid fixation decreased the recurrence rate and achieved satisfactory outcomes at mid to long term follow-up [16]. Based on the findings of this review, severe varus deformity is successfully and satisfactorily corrected by DLO.

### Study limitations

Limitations of this study include the relatively low volume of data collected and the number of studies included.

## Conclusion

DLO showed a low complication rate and satisfactory short term KOOS and IKDAC scores. Randomised control trials with long term follow-up comparing the DLO and HTO are recommended to further compare the two options.

## Conflict of interest

The authors declare that they have no relevant financial or non-financial interests to report.

## Funding

This research did not receive any specific funding.

## Ethical approval

Ethical approval was not required.

## Informed consent

This article does not contain any studies involving human subjects.

## Authors’ Contributions

All authors had made impactful contributions to the manuscript submitted. The first author was involved in selecting the included studies during the process of screening, study design, statistical analysis and drafting of the review. The second author was involved with data extraction, data analysis and interpretation, the third and the fourth authors played an essential role in this study, providing necessary guidance and mentorship.

## References

[1] Hoorntje A, Kuijer PPFM, Koenraadt KLM, et al. (2020) Return to sport and work after randomization for knee distraction versus high tibial osteotomy: Is there a difference? Epub ahead of print. Erratum for: J Knee Surg. PMID: 33853157. 10.1055/s-0040-1721027. 33231278

[2] Liu JN, Agarwalla A, Garcia GH, et al. (2019) Return to sport and work after high tibial osteotomy with concomitant medial meniscal allograft transplant. Arthrosc – J Arthrosc Relat Surg 35, 3090–3096. 10.1016/j.arthro.2019.05.053 31699261

[3] Puzzitiello RN, Liu JN, Garcia GH, et al. (2020) Return to work after distal femoral varus osteotomy. Orthop J Sport Med 8, 2325967120965966. 10.1177/2325967120965966 PMC7720305 33330734

[4] Nakayama H, Kanto R, Onishi S, et al. (2021) Cartilage repair examined by second-look arthroscopy following double-level osteotomy performed for osteoarthritic knees with severe varus deformity. Knee 29, 411–417. 33714928 10.1016/j.knee.2021.02.024

[5] Lobenhoffer P, Kley K, Freiling D, van Heerwaarden R (2017) Medial closed wedge osteotomy of the distal femur in biplanar technique and a specific plate fixator. Oper Orthop Traumatol 29, 306–319. 28497247 10.1007/s00064-017-0493-9

[6] Niemeyer P, Koestler W, Kaehny C, et al. (2008) Two-year results of open-wedge high tibial osteotomy with fixation by medial plate fixator for medial compartment arthritis with varus malalignment of the knee. Arthroscopy, 24(7), 796–804. 18589268 10.1016/j.arthro.2008.02.016

[7] Babis GC, An KN, Chao EYS, et al. (2002) Double level osteotomy of the knee: A method to retain joint-line obliquity clinical results. J Bone Jt Surg – Ser A 84, 1380–1388. 10.2106/00004623-200208000-00013 12177268

[8] Jud L, Vlachopoulos L, Beeler S, et al. (2020) Accuracy of three dimensional-planned patient-specific instrumentation in femoral and tibial rotational osteotomy for patellofemoral instability. Int Orthop 44, 1711–1717. 32055971 10.1007/s00264-020-04496-y

[9] Saragaglia D, Nemer C, Collew PE (2008) Computer-assisted double level osteotomy for severe genu varum. Sports Med Arthrosc 16, 91–96. 18480728 10.1097/JSA.0b013e318172b562

[10] Strecker W. 2007. Planning analysis of knee-adjacent deformities: I. Frontal plane deformities. Eur J Trauma Emerg Surg 33, 662–668. 26815097 10.1007/s00068-007-5175-0

[11] Hernigou P, Queinnec S, Picard L, et al. (2013) Safety of a novel high tibial osteotomy locked plate fixation for immediate full weight-bearing: A case-control study. Int Orthop 37, 2377–2384. 23974839 10.1007/s00264-013-2066-3 PMC3843216

[12] Hofmann S, Lobenhoffer P, Staubli A, Van Heerwaarden R (2009) Osteotomies of the knee joint in patients with monocompartmental arthritis. Orthopade 38, 755–770. 19629433 10.1007/s00132-009-1458-y

[13] Ehlinger M, D’Ambrosio A, Vie P, et al. (2017) Total knee arthroplasty after opening– versus closing-wedge high tibial osteotomy. A 135-case series with minimum 5-year follow-up. Orthop Traumatol Surg Res 103, 1035–1039. 28888524 10.1016/j.otsr.2017.07.011

[14] Preston S, Howard J, Naudie D, et al. (2014) Total knee arthroplasty after high tibial osteotomy, no differences between medial and lateral osteotomy approaches. Springer: New York LLC. 10.1007/s11999-013-3040-5 PMC3889445 23657880

[15] Ramappa M, Anand S, Jennings A (2013) Total knee replacement following high tibial osteotomy versus total knee replacement without high tibial osteotomy: a systematic review and meta analysis. Springer Verlag. 10.1007/s00402-013-1838-y 23959070

[16] Nakayama H, Schröter S, Yamamoto C, et al. (2018) Large correction in opening wedge high tibial osteotomy with resultant joint-line obliquity induces excessive shear stress on the articular cartilage. Springer Verlag. 10.1007/s00167-017-4680-x 28831525

[17] Hernigou P, Giber D, Dubory A, Auregan JC (2020) Safety of simultaneous versus staged bilateral opening-wedge high tibial osteotomy with locked plate and immediate weight bearing. Int Orthop 44, 109–117. 31385014 10.1007/s00264-019-04385-z

[18] Angel JC, Liyanage SP, Griffiths WEG (1974) Double osteotomy for the relief of pain in arthritis of the knee. Rheumatology 13, 109–119. 10.1093/rheumatology/13.3.109 4421756

[19] Benjamin A (1969) Double osteotomy for the painful knee in rheumatoid arthritis and osteoarthritis. J Bone Joint Surg Br 51, 694–699. 5371973

[20] Benjamin A (1974) Double osteotomy of the knee. Scand J Rheumatol 3, 65. 4421757

[21] Schüller HM, van Dijk CN, Fidler MW (1987) Poor results of double osteotomy for the rheumatoid knee. Acta Orthop 58, 253–255. 10.3109/17453678709146478 3630656

[22] Iveson JMI, Longton EB, Wright V (1977) Comparative study of tibial (single) and tibiofemoral (double) osteotomy for osteoarthrosis and rheumatoid arthritis. Ann Rheum Dis 36, 319–326. 901030 10.1136/ard.36.4.319 PMC1006692

[23] Zaalberg GS, Wouters HW (1972) Double osteotomy of the knee-joint according to Benjamin. Acta Orthop Belg 58, 89–90. 5049405

[24] Schröter S, Nakayama H, Yoshiya S, et al. (2019) Development of the double level osteotomy in severe varus osteoarthritis showed good outcome by preventing oblique joint line. Arch Orthop Trauma Surg 139, 519–527. 30413943 10.1007/s00402-018-3068-9

[25] Nakayama H, Iseki T, Kanto R, et al. (2020) Physiologic knee joint alignment and orientation can be restored by the minimally invasive double level osteotomy for osteoarthritic knees with severe varus deformity. Knee Surgery, Sport Traumatol Arthrosc 28, 742–750. 10.1007/s00167-018-5103-3 30196434

[26] Ji W, Luo C, Zhan Y, et al. (2019) A residual intra-articular varus after medial opening wedge high tibial osteotomy (HTO) for varus osteoarthritis of the knee. Arch Orthop Trauma Surg 139, 743–750. 30673869 10.1007/s00402-018-03104-4

[27] Bin Abd Razak HR, Jacquet C, Wilson AJ, et al. (2021) Minimally invasive high tibial osteotomy using a patient-specific cutting guide. Arthrosc Tech 10, e431–e435. 33680776 10.1016/j.eats.2020.10.029 PMC7917192

[28] Paley D, Herzenberg JE, Tetsworth K, McKie J, Bhave A (1994) Deformity planning for frontal and sagittal plane corrective osteotomies. Orthop Clin North Am 25, 425–465. 8028886

[29] Schuster P, Geßlein M, Schlumberger M, et al. (2018) Ten-year results of medial open-wedge high tibial osteotomy and chondral resurfacing in severe medial osteoarthritis and varus malalignment. Am J Sports Med 46, 1362–1370. 29589953 10.1177/0363546518758016

[30] Saragaglia D, Mercier N, Colle PE (2010) Computer-assisted osteotomies for genu varum deformity: Which osteotomy for which varus? Int Orthop 34, 185–190. 19305996 10.1007/s00264-009-0757-6 PMC2899360

[31] Dugdale TW, Noyes FR, Styer D (1992) Preoperative planning for high tibial osteotomy. The effect of lateral tibiofemoral separation and tibiofemoral length. Clin Orthop Relat Res 274, 248–264. 1729010

[32] Miniaci A, Ballmer FT, Ballmer PM, Jakob RP (1989) Proximal tibial osteotomy. A new fixation device. Clin Orthop Relat Res 250–259. 2766613

[33] Feucht MJ, Winkler PW, Mehl J, et al. (2021) Isolated high tibial osteotomy is appropriate in less than two-thirds of varus knees if excessive overcorrection of the medial proximal tibial angle should be avoided. Sport Traumatol Arthrosc 29, 3299–3309. 10.1007/s00167-020-06166-3 PMC8458209 32691093

[34] Julian PT, Higgins JT, Chandler J, Cumpston M, Li T, Page MJ, Welch VA (2019) Cochrane handbook for systematic reviews of interventions, 2nd edn John Wiley & Sons: Chichester, UK.

[35] Akaoka Y, Iseki T, Kanto R, et al. (2020) Changes in patellar height and patellofemoral alignment following double level osteotomy performed for osteoarthritic knees with severe varus deformity. Asia-Pacific J Sport Med Arthrosc Rehabil Technol 22, 20–26. 10.1016/j.asmart.2020.05.003 PMC7369356 32728526

[36] Fürmetz J, Patzler S, Wolf F, et al. (2020) Tibial and femoral osteotomies in varus deformities – radiological and clinical outcome. BMC Musculoskelet Disord 21, 201. 32234018 10.1186/s12891-020-03232-2 PMC7110680

[37] Hai H, Takahashi I, Shima N, et al. (2020) Preliminary evaluation of the efficacy of postoperative early weight-bearing rehabilitation protocol for patients after double-level osteotomy. Prog Rehabil Med 5, 20200017. 32844130 10.2490/prm.20200017 PMC7429558

[38] Saragaglia D, Rubens-Duval B, Chaussard C (2007) Computer-assisted combined femoral and tibial osteotomy for severe genu varum: Early results in 16 patients. Rev Chir Orthop Reparatrice Appar Mot 93, 351–356. 17646816 10.1016/s0035-1040(07)90276-7

[39] Saito H, Yonekura A, Saito K, et al. (2021) A new double level osteotomy procedure to restore a joint line and joint angles in severe varus osteoarthritis. – Double level osteotomy associated with tibial condylar valgus osteotomy (DLOTO). Asia-Pacific J Sport Med Arthrosc Rehabil Technol 24, 9–13. 10.1016/j.asmart.2020.11.001 PMC7787959 33457209

[40] Saragaglia D, Blaysat M, Mercier N, Grimaldi M (2012) Results of forty two computer-assisted double level osteotomies for severe genu varum deformity. Int Orthop 36, 999–1003. 21947287 10.1007/s00264-011-1363-y PMC3337108

[41] Akamatsu Y, Nejima S, Tsuji M, et al. (2021) Joint line obliquity was maintained after double-level osteotomy, but was increased after open-wedge high tibial osteotomy. Knee Surg Sport Traumatol Arthrosc 30(2), 688–697. 10.1007/s00167-020-06430-6 33433634

[42] Akamatsu Y, Kumagai K, Kobayashi H, et al. (2018) Effect of increased coronal inclination of the tibial plateau after opening-wedge high tibial osteotomy. Arthrosc – J Arthrosc Relat Surg 34, 2158–2169.e2. 10.1016/j.arthro.2018.01.055 29685834

[43] Goshima K, Sawaguchi T, Shigemoto K, et al. (2019) Comparison of Clinical and radiologic outcomes between normal and overcorrected medial proximal tibial angle groups after open-wedge high tibial osteotomy. Arthrosc – J Arthrosc Relat Surg 35, 2898–2908.e1. 10.1016/j.arthro.2019.04.030 31604511

[44] Goshima K, Sawaguchi T, Shigemoto K, et al. (2019) Large opening gaps, unstable hinge fractures, and osteotomy line below the safe zone cause delayed bone healing after open-wedge high tibial osteotomy. Knee Surgery, Sport Traumatol Arthrosc 27, 1291–1298. 10.1007/s00167-018-5334-3 30539305

[45] Lee KM, Chang CB, Park MS, et al. (2015) Changes of knee joint and ankle joint orientations after high tibial osteotomy. W.B. Saunders Ltd. 10.1016/j.joca.2014.11.001 25450843

[46] Oh KJ, Ko YB, Bae JH, et al. (2016) Analysis of knee joint line obliquity after high tibial osteotomy. J Knee Surg 29, 649–657. 26838969 10.1055/s-0036-1571430

[47] Halder A, Kutzner I, Graichen F, et al. (2012) Influence of limb alignment on mediolateral loading in total knee replacement. J Bone Jt Surg 94, 1023–1029. 10.2106/JBJS.K.00927 22637208

[48] Saragaglia D, Chedal-Bornu B, Rouchy RC, et al. (2016) Role of computer-assisted surgery in osteotomies around the knee. Knee Surg Sport Traumatol Arthrosc 24, 3387–3395. 10.1007/s00167-016-4302-z 27585448

